# Biological processes and signal transduction pathways regulated by the protein methyltransferase SETD7 and their significance in cancer

**DOI:** 10.1038/s41392-018-0017-6

**Published:** 2018-07-13

**Authors:** Inês de Albuquerque Almeida Batista, Luisa Alejandra Helguero

**Affiliations:** Institute for Biomedicine (iBiMED), Department of Medical Sciences, University of Aveiro, Aveiro, Portugal

## Abstract

Protein methyltransferases have been shown to methylate histone and non-histone proteins, leading to regulation of several biological processes that control cell homeostasis. Over the past few years, the histone-lysine *N*-methyltransferase SETD7 (SETD7; also known as SET7/9, KIAA1717, KMT7, SET7, SET9) has emerged as an important regulator of at least 30 non-histone proteins and a potential target for the treatment of several human diseases. This review discusses current knowledge of the structure and subcellular localization of SETD7, as well as its function as a histone and non-histone methyltransferase. This work also underlines the putative contribution of SETD7 to the regulation of gene expression, control of cell proliferation, differentiation and endoplasmic reticulum stress, which indicate that SETD7 is a candidate for novel targeted therapies with the aim of either stimulating or inhibiting its activity, depending on the cell signaling context.

## Cancer therapeutics: a context-dependent target

The involvement of the methyltransferase SETD7 in cell proliferation, differentiation and endoplasmic reticulum (ER) stress makes it a promising therapeutic target for cancer. By transferring methyl groups to lysine residues on DNA packaging proteins, histones, as well as on over 30 non-histone proteins, SETD7 regulates gene expression and influences protein function and stability. Inês de Albuquerque Almeida Batista and Luisa A. Helguero at the University of Aveiro, Portugal, review the latest knowledge on SETD7 activity highlighting its tissue-specific effects on cell differentiation and proliferation and its ability to promote the transcription of genes that mitigate ER stress. Further understanding SETD7’s effects in disease conditions will help determine whether stimulating or inhibiting SETD7 activity in particular cell signaling contexts represents an effective approach for the treatment of cancer.

## Introduction

Gene expression is regulated by different mechanisms, such as alterations of chromatin conformation and transcription factor activity.^1,2^ These processes are regulated by protein posttranslational modifications (PTMs). Histone PTMs modulate gene transcription^3–5^ as they regulate the accessibility of transcription factors and chromatin remodelers by inducing euchromatin or a heterochromatic conformational state.^1^ Histone methyltransferases (HMTs) can mediate transcriptional activation or suppression depending on the histone targeted and amino-acid position.^5,6^ For instance, histone H3 methylation at lysine 4 (H3K4me) is associated with transcriptional activation, whereas H3K9me3 is found in silenced chromatin.^6–8^ HMTs have been recently shown to methylate non-histone substrates (e.g., transcription factors and transcriptional co-regulators).^9^ In general, the addition of methyl groups to non-histone substrates can affect their stability (inhibiting or stimulating their degradation), subcellular localization, and protein–protein interactions or even influence the occurrence of additional protein modifications. In addition, the affinity of transcription factors to the promoter of their target genes can be enhanced/diminished upon methylation of these factors, leading to transcription activation or repression.^10^

SETD7 (also known as SET7/9, KIAA1717, KMT7, SET7, SET9) is a lysine methyltransferase (KMT) that methylates H3K4.^11,12^ SETD7 contains a Su(var)3-9, Enhancer-of-zeste and Trithorax (SET) domain that is responsible for the transfer of a methyl group to a lysine residue of various substrates. SETD7 histone and non-histone substrates are involved in distinct cellular processes, for instance, in cell cycle regulation, the DNA damage response, RNA polymerase II-dependent gene transcription, chromatin modulation and cell differentiation. Hence, SETD7 plays a critical role in several physiological and pathological processes.^13–15^

This review presents the current knowledge of the structure, function and cellular distribution of SETD7 and describes its role in specific cellular processes, such as proliferation and differentiation. This review also describes the main pathways and molecules that are methylated by or cooperate with SETD7, as well as those that regulate SETD7 activity and cellular localization. Finally, the significance of SETD7 in the endoplasmic reticulum (ER) stress response and in cancer development and progression are discussed, and the potential of this enzyme for targeted cancer treatment is highlighted.

## SETD7 structure and function

**Fig. 1:**
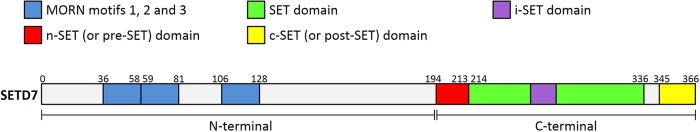
Schematic representation of the SETD7 domains. The N-terminal part of SETD7 is responsible for its stabilization and possibly its intracellular localization. The N-terminal part of SETD7 contains three MORN motifs, which normally mediate protein binding to plasma membrane phospholipids. The C-terminus is responsible for the catalytic function of SETD7 and binding to its substrates and cofactor SAM. Thus, the C-terminus contains the SET domain, which is flanked by the n-SET (or pre-SET) and c-SET (or post-SET) domains and contains an insertion region called i-SET. This SET domain is highly conserved among all methyltransferases. MORN membrane occupation and recognition nexus, SAM S-adenosylmethionine

SETD7 is a 41-kDa lysine monomethyltransferase that is responsible for the methylation of various histone and non-histone substrates. Similar to most KMTs, SETD7 contains a SET domain that is responsible for catalysis of the cofactor S-adenosylmethionine (SAM) and the subsequent transfer of a methyl group to a lysine residue.^3,7,13^ SETD7 is mainly composed of several β strands that are organized in two domains: the N-terminal domain and C-terminal domain (or SET domain), as shown in Fig. 1. The N-terminal domain is a non-conserved domain formed by a series of antiparallel β strands. The C-terminal domain is a globular structure formed by β strands that are organized into three discrete sheets surrounding a pseudo-knot (a knot-like structure formed by the C-terminal segment of the SET domain passing through a loop structure made by the preceding segment of the SET domain). The SET domain is highly conserved among almost all methyltransferases and is responsible for their catalytic function.^16,17^ This domain is flanked by a set of variable regions, pre-SET (or n-SET), i-SET and post-SET (or c-SET), which are inserted immediately N-terminally within the SET domain or immediately C-terminally outside of the SET domain (Fig. 1). An unusual feature of SET domain-containing methyltransferases is that the substrate and cofactor SAM bind distinct sites on opposite surfaces of the SET domain,^17–19^ in contrast to other methyltransferase structures.^20^ SETD7 has an ordered, sequential binding mechanism in which SAM binds first to the enzyme, followed by substrate binding.^21^ Both the i-SET and post-SET regions along with the SET domain contribute to form the substrate and cofactor binding sites, as well as a narrow hydrophobic channel, through which the substrate lysine and cofactor meet at the enzyme´s core. This structure shields the lysine from solvent and is essential for catalysis, as well as the transfer of a methyl group from SAM to the ε-nitrogen (previously deprotonated) of the substrate target lysine.^17,18^ On the other hand, the pre-SET region and N-terminal domain stabilize the enzyme and the SET domain.^16,17^ Pre-SET is also a binding interface to other proteins or DNA.^18^ Additionally, the N-terminal domain seems to provide a portion of the binding pocket for the histone N-terminal tail.^16^

SETD7 was originally isolated by Wang et al. as a H3K4-specific methyltransferase.^11,12^ In general, H3K4 methylation is a marker of transcriptional activation. This distinctive marker on H3K4 is often found near enhancer binding sites and the transcription start site (TSS) of actively transcribed genes^22–25^ and is recognized by “reader” proteins/effectors, which then stimulate transcription of specific genes.^7,26^ SETD7 can also monomethylate non-histone substrates, such as p53,^27^ TAF10 ^28^ and estrogen receptor alpha (ERα).^29^ Some studies have also identified SETD7 as a di-methyltransferase. For example, Dhayalan et al. confirmed that SETD7 transfers two methyl groups to Msx2-interacting nuclear target protein (MINT) via mass spectrometry. The H3 histone was also dimethylated by SETD7, but at a lower efficiency (∼10%).^30^ Nevertheless, structural studies show that the free-energy barrier is 5 kcal/mol higher for the second methyl transfer to H3K4me in comparison with the barrier for monomethylation by SETD7 (which is ≈ 17–18 kcal/mol). These barriers decrease significantly for the SETD7-Y305F mutant. Therefore, SETD7 should be considered to be a monomethyltransferase.^31–33^ Future studies should clarify this issue. One explanation that has been suggested is that the methyl group added by SETD7 may be recognized by other methyltransferases and therefore acts as a platform for the subsequent addition of a second and third methyl group by other methyltransferases.^13,30,34^

SETD7 methylates substrates that possess the conserved Lysine/Arginine-Serine/Threonine-Lysine* (K/R-S/T-K*) consensus motif (with K* as the target lysine).^9,17,30^ Substrate recognition is mediated by the SET, i-SET and post-SET domains.^17^ Polar electrostatic interactions also seem to be involved, bringing together the electropositive substrate and correspondent electronegative binding site.^17,18^

A selective inhibitor of SETD7 methyltransferase activity—(R)-PFI-2—was recently developed. (R)-PFI-2 occupies the substrate lysine-binding groove of SETD7, competing with the substrate and preventing its binding to SETD7. In addition, the pyrrolidine moiety of (R)-PFI-2 forms hydrophobic interactions with the departing methyl group of the cofactor SAM, which may explain the cofactor-dependent inhibitory mechanism of (R)-PFI-2. Specifically, (R)-PFI-2 binds to SETD7 only after SAM binds to the enzyme.^35^ This compound will be useful for future studies aimed at defining and better understanding the function of SETD7 and will have the potential for targeted interventions.

## SETD7 cellular localization

SETD7 targets can either be found in the nucleus or the cytoplasm,^13,14,36–38^ which implies nuclear and cytoplasmic localization for SETD7. However, in contrast to other SET domain KMTs, SETD7 does not carry nuclear localization and export signals, which would most likely lead to a stronger cytoplasmic localization.^39^ The SETD7 cellular localization may be regulated by other cellular factors; this hypothesis is supported by the observation that nuclear factor (NF)-kappa-B (NFκB) recruits SETD7 to the promoters of NFκB-dependent genes.^37^ The SETD7 localization may also depend on the cell type and cells’ specific needs and functions. For example, although in mouse embryonic fibroblasts (MEFs), SETD7 retains Yes-associated protein (YAP) in the cytoplasm;^40^ in human monocytes, SETD7 colocalizes with NFκB-p65 both in the cytoplasm and nucleus.^37^ However, there is not enough evidence to support the tethered recruitment hypothesis to explain the SETD7 nuclear localization, and further research needs to be conducted to elucidate how SETD7 is transported into the nucleus. Additionally, the SETD7 distribution within cells can be influenced by extracellular factors and cell exposure to different hazards. SETD7 accumulates in the nucleus of endothelial cells in response to transcriptional inhibition by actinomycin D or when these cells are exposed to a high glucose level.^38^

SETD7 contains membrane occupation and recognition nexus (MORN) motifs (Fig. 1), which may act as protein-phospholipid binding domains and mediate the interaction and anchorage of a protein to the plasma membrane.^41,42^ To date, protein substrates located in the membrane have not been identified. Taken together, these observations suggest that SETD7 has pleiotropic effects throughout the cell.

## SETD7 substrates and their biological effects

**Fig. 2:**
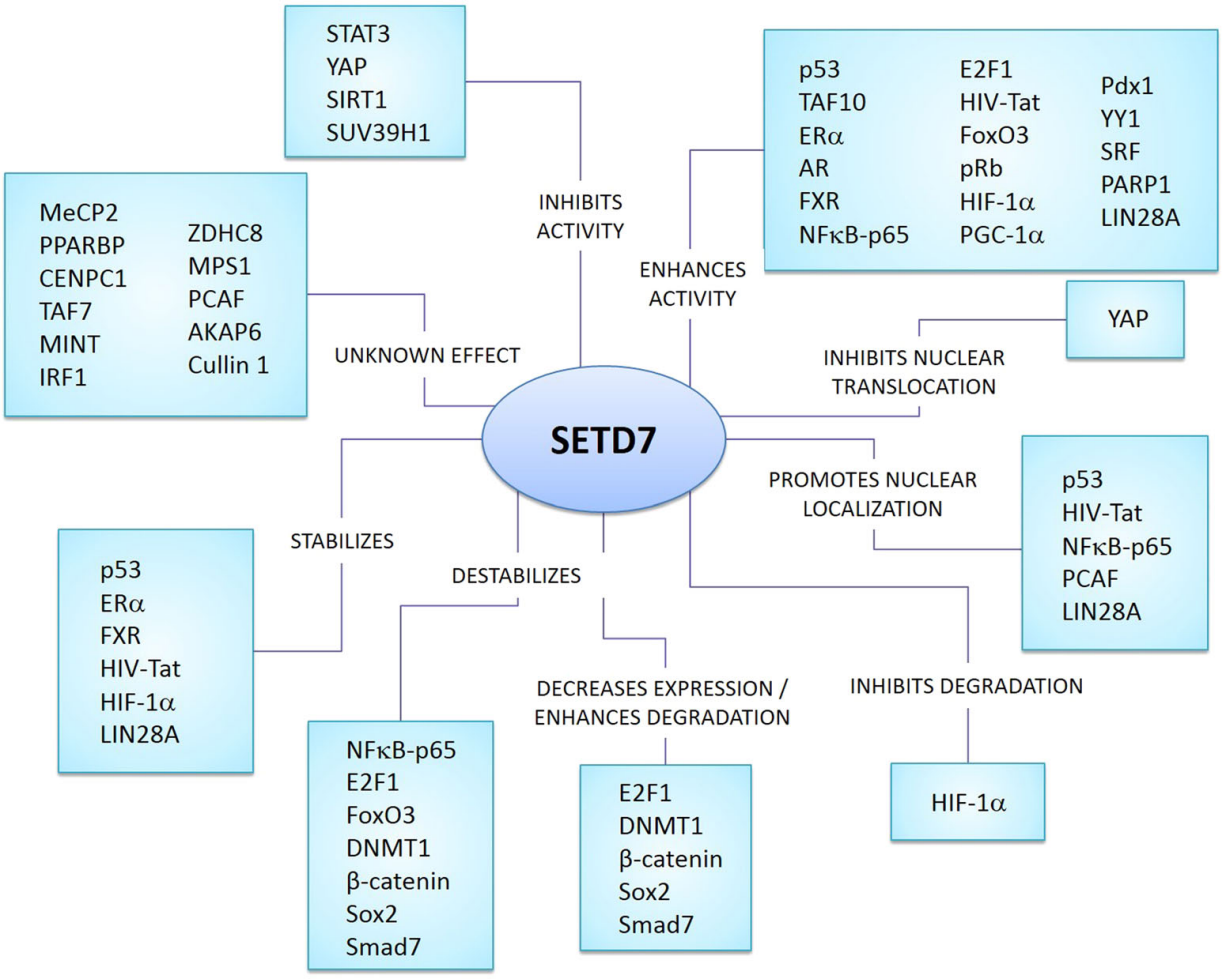
Effects of SETD7-mediated methylation on its non-histone substrates. SETD7 methylates >30 non-histone substrates, which are associated with a broad spectrum of cellular processes. Through methylation, SETD7 is able to regulate the function, degradation and intracellular localization of these proteins

**Fig. 3:**
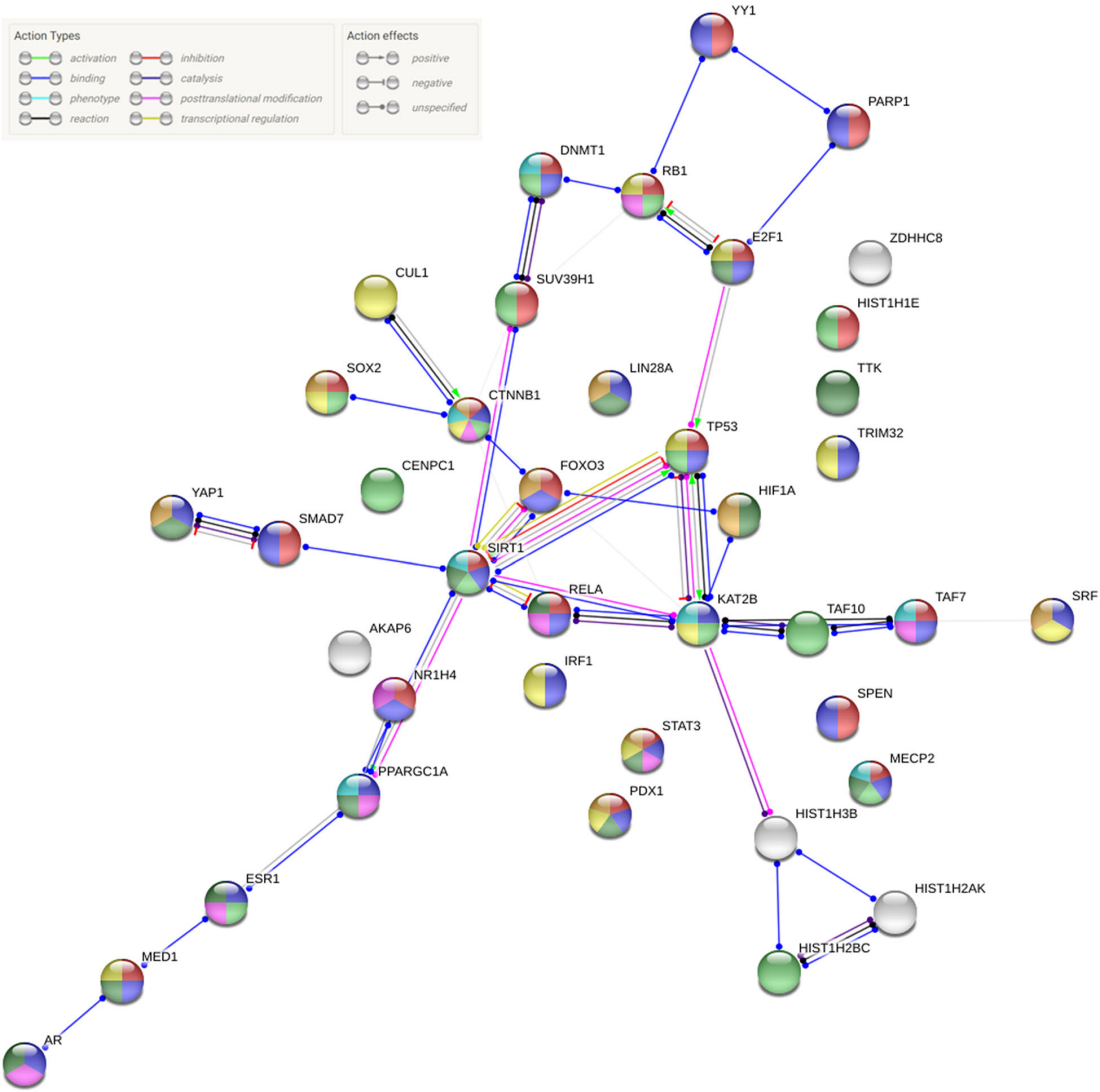
Interaction network between known SETD7 target proteins showing the biological processes regulated. Protein interactions and biological processes were analyzed using the online tool STRING (https://string-db.org). Red: negative regulation of transcription from RNA polymerase II promoter (FDR = 3.73e-17); dark violet: positive regulation of gene expression (FDR = 7.82e-17); light green: chromatin organization (FDR = 5.93e-10); light violet: intracellular receptor signaling pathway (FDR = 8.49e-9); dark green: positive regulation of cell proliferation (FDR = 9.59e-9); yellow: negative regulation of cell proliferation (FDR = 1.11e-8); light blue: regulation of histone modification (FDR = 2.7e-7); and beige: stem cell differentiation (FDR = 3.81e-7). The action type and effects depict the type of regulation between two proteins

SETD7 methylates an ample array of substrates, of which histones and transcriptional co-regulators are over-represented (Table 1 and Fig. 2).^11,12,36,43^ SETD7 substrates are involved in distinct cellular processes, including cell cycle regulation, the DNA damage response, RNA polymerase II-dependent gene transcription, chromatin modulation and cell differentiation,^13,14^ and interact with each other to form a complex network that is regulated through protein–protein interactions (Fig. 3). Hence, SETD7 may play a critical role in several physiological and pathological processes,^15^ such as metabolism, immunity and cancer.

**Table 1:** SETD7 histone and non-histone substrates

SETD7 targets	Lysine residues methylated	Domain/region of methylation^a^	References
AKAP6	K604	Unknown	^30^
AR	K630	DNA-binding domain	^36,89^
β-Catenin	K180	ARM 1 repeat	^70^
CENPC1	K414	Unknown	^30^
Cullin 1	K73	Unknown	^30^
DNMT1	K142	Unknown	^36,77,81^
ERα	K302	Hinge domain	^29,89^
E2F1	K185	DNA-binding domain	^96–98^
FoxO3	K271	Nuclear localization signal	^103,108^
FXR	K206	DNA-binding domain	^116,117^
HIF-1α	K32	Basic helix-loop-helix domain	^124–126^
Histone 1.4	K34, K121, K129, K159, K171, K177 and K192	N-terminal (K34) and C-terminal domains	^36,62,63^
Histone 2A	Unknown	Unknown	^30,36^
Histone 2B	Unknown	Unknown	^30,36^
Histone 3	K4	N-terminal tail	^11,12,44^
HIV-Tat	K51, K71	RNA-binding (K51) and glutamine-rich (K71) domains	^118,121^
IRF1	K126	Nuclear localization signal	^30^
LIN28A	K135	Flexible linker region	^131^
MeCP2	K347	C-terminal domain	^30,36^
MINT	K2076	Unknown	^30^
NFκB-p65	K37, K314, K315	RelA homology domain (K37)	^137,138^
p53	K372	Negative regulatory domain	^27,142–144^
PARP1	K508	Automodification domain	^147^
PCAF	K78, K89, K638, K671, K672 and K692	*N*-acetyltransferase domain (K638)	^163^
Pdx1	K123 and K131	Antp-type hexapeptide motif (K123)	^171,172^
PGC-1α	K779	Unknown	^176^
PPARBP or Med1	K1006	Unknown	^30^
pRb	K810 and K873	Domain C (K810 and K873) and nuclear localization signal (K873)	^100,101^
SIRT1	K232, K235, K236 and K238	Unknown	^36,179^
Smad7	K70	MH1 domain	^188^
Sox2	K119	Unknown	^193^
SRF	Unknown	Unknown	^194^
STAT3	K140	Coiled-coil domain	^199,200^
SUV39H1	K105 and K123	Unknown	^203^
TAF10	K189	Histone fold domain	^28^
TAF7	K5	Unknown	^36,199,206^
TTK or MPS1	K708	Protein kinase domain	^30^
YAP	K494	Transactivation domain	^35,40^
YY1	K173 and K411	Transcription repression (K173) and DNA-binding (K411) domains	^212^
ZDHHC8	K300	Unknown	^30^

^a^According to the Uniprot and Conserved Domain database at NCBI

## SETD7 histone targets

SETD7 methylates H3 at lysine 4, which enhances transcriptional activation by preventing chromatin condensation.^11,12,44^ The H3K4me1 modification is often found near enhancer binding sites and the TSS of actively transcribed genes;^22–25^ H3K4me1 is recognized by “reader” proteins that are then recruited to local chromatin to stimulate the transcription of specific genes.^7,26^ The genes regulated by this modification are currently being identified. However, SETD7-dependent methylation of H3K4 has been shown to be associated with transcriptional activation of myogenic differentiation genes, such as *MYOD*, *MYOGENIN*, *MHC* and *MCK*.^15^ SETD7 may also regulate oxidative stress and inflammation as H3K4me1 was found to be enriched at the promoter of *RELA/NFκB-p65* in response to reactive oxygen species (ROS) stimulation, thereby promoting NFκB production.^45^ SETD7 was also shown to favor mono- and dimethylation of H3K4 at the promoter of *NOS2* in response to cytokines in β cells, increasing nitric oxide production and cytokine-induced apoptosis.^46^ H3K4 is methylated in β-cell-specific genes, such as *INS1/2*, which regulates insulin secretion and glucose metabolism.^34,46^ Akiyama et al. found that SETD7 may act as a tumor suppressor in gastric cancer through H3K4 monomethylation at the *SREK1IP1* TSS region and at the *PGC* and *CCDC28B* promoter regions, which are involved in the regulation of splicing, cell differentiation and proliferation, respectively.^47^ Moreover, H3K4 methylation was found in other genes that are important for the regulation of cell proliferation, differentiation and apoptosis. However, it is unclear whether SETD7 is responsible for H3K4 methylation of these genes.^7,48^ One example of genes that are regulated by H3K4 methylation are *HOX* genes,^49–51^ which are mostly known for their role in embryonic development, coordinating tissue-specific cell growth, proliferation and differentiation.^52–55^ In adulthood, *HOX* regulates cell renewal,^56^ hematopoiesis,^57^ cell fate, differentiation and tissue homeostasis.^55,58,59^ *HOX* expression dysregulation has been associated with cancer. Interestingly, *HOX* can either be upregulated or downregulated in cancer and either have an oncogenic or a tumor-suppressor effect depending on the tissue context and cancer type.^59–61^ Although it is thought that H3K4 methylation at the promoters of *HOX* is mediated by mixed lieneage leukemia  methyltransferases, the possibility that SETD7 may also contribute to *HOX* transactivation has yet to be studied.^49–51^

SETD7 was reported to methylate histones H2A and H2B.^30,36^ However, SETD7 does not methylate H2A and H2B when they are confined within chromatin.^11^ Therefore, it is hypothesized that only free H2A and H2B are methylated by SETD7. The specific lysine residues targeted and functional effect of this modification are still unknown.^30,36^ In addition, SETD7 methylates the linker histone variant H1.4 at seven different lysine residues (K34, K121, K129, K159, K171, K177 and K192), which can influence H1 binding to DNA and its function in chromatin compaction.^36,62,63^

## SETD7 non-histone substrates

### A-kinase anchor protein 6

A-kinase anchor protein 6 (AKAP6) is a scaffolding protein that binds and targets signaling enzymes to specific locations, facilitating enzyme–substrate interactions.^64,65^ All of the members of the AKAP family contain a protein kinase A (PKA)-binding domain and play a role in the cyclic adenosine monophosphate signaling pathway.^66^ AKAP6 is involved in the regulation of myocardial contractility and hypertrophy, muscle regeneration and myogenic differentiation.^67^ Dhayalan et al. reported that SETD7 catalyzes AKAP6 methylation at lysine K604. Nevertheless, little is still known about the effects exerted by AKAP6 methylation^30^ or the K604 function in the AKAP6 structure.

### β-Catenin

This dual-function protein is part of the plasma membrane adherens junction complex, which mediates cell–cell adhesion and epithelial integrity, and is a downstream effector of the Wnt signaling pathway. Upon Wnt binding to its receptor, cytoplasmic β-catenin degradation by glycogen synthase kinase-3 beta (GSK3β) is inhibited, and β-catenin enters the nucleus and promotes transcription initiation of genes that are involved in cell fate decisions, including proliferation, migration and invasion.^68,69^ SETD7 methylates β-catenin at K180, which strengthens the interaction of β-catenin with GSK3β. GSK3β phosphorylates β-catenin, which leads to ubiquitination and proteasomal degradation. SETD7 knockdown results in an increase in HeLa cell proliferation, which is reversed by β-catenin knockdown. These results indicate that SETD7 indirectly decreases cell proliferation by targeting β-catenin for degradation and preventing the transcription of its downstream target genes.^70^ However, Oudhoff et al. found that SETD7 contributes to β-catenin nuclear translocation upon Wnt activation or GSK3 inhibition, with evidence suggesting that this translocation results from the interaction of methylated YAP (by SETD7, see below) and β-catenin.^71^ Whether these discrepancies are related to the cell type and context remains to be elucidated.

### Centromere protein C1

Centromere protein C1 (CENPC1) associates with centromeric DNA and assists in the assembly of kinetochores to centromeres. Therefore, CENPC1 is essential for the correct segregation of sister chromatids toward the spindle poles during cell division.^72,73^ CENPC1 K414 monomethylation is catalyzed by SETD7.^30^ Little is known about the biological effects of this modification; therefore, further studies are required to determine how SETD7 influences the function of CENPC1.

### Cullin 1

This is a scaffold protein of the largest family of E3 ubiquitin ligases, the Skp1/Cullin 1/Rbx1/F-box protein (SCF) complex, which is required for ubiquitin-dependent degradation of proteins that are important for cell cycle regulation, signal transduction and transcription.^74,75^ Cullin 1 promotes cell cycle progression through ubiquitination of p27 and p21 and plays an important role in early embryogenesis.^76^ Therefore, Cullin 1 dysregulation can increase protein ubiquitination and degradation, as well as lead to uncontrolled cell cycle progression and embryonic lethality. Furthermore, Cullin 1 was found to be upregulated in breast and gastric cancer, contributing to a poor prognostic due to increased cell proliferation, migration and invasion.^74–76^ Cullin 1 methylation by SETD7 occurs at K73, but further studies are needed to better understand and identify the SETD7 effects over Cullin 1 expression and function.^30^

### DNA cytosine-5-methyltransferase 1

DNA cytosine-5-methyltransferase 1 (DNMT1) is a maintenance DNA methyltransferase that methylates DNA at hemimethylated CpG islands, which results in transcriptional repression.^77,78^ DNMT1 also represses gene expression by recruiting and interacting with histone deacetylases (HDACs) and other transcriptional inhibitors.^79,80^ DNMT1 methylation at K142 by SETD7 correlates with a decrease in DNMT1 by facilitating ubiquitination and proteasome-mediated degradation.^36,77^ Moreover, DNMT1 expression is regulated by two mutually exclusive PTMs: K142 monomethylation by SETD7 (which promotes DNMT1 degradation) and S143 phosphorylation by AKT1 (which stabilizes DNMT1). These PTMs lead to gene repression/activation, respectively, during the cell cycle and development.^81^ DNMT1 dysregulation is associated with the silencing of tumor-suppressor genes,^80^ and DNMT1 was found to be overexpressed and associated with poor prognosis in prostate,^82^ breast,^83^ colon,^84^ gastric,^85^ bladder^86^ and lung^87^ cancers. Thus, DNMT1 methylation by SETD7 can be addressed as an interesting process to be explored in the development of new therapeutic strategies, particularly for cancer treatment.

### ERα and AR

ERα and androgen receptor (AR) are nuclear receptors that, once activated, are translocated into the nucleus, where they function as transcriptional factors. ERα and AR are essential for the regulation of differentiation and proliferation of breast and prostate cells, respectively.^29,88^ ERα stimulates transcription of *CYCLIN D1* and anti-apoptotic genes. Approximately two-thirds of breast cancers are ERα-positive and will potentially respond well to anti-estrogens.^29,43^ SETD7-driven methylation at ERα-K302 positively affects the stability of the ERα protein and transcriptional efficiency of the ERα gene.^29^ SETD7 knockdown leads to a twofold decrease of the ERα half-life, and K302 methylation stabilizes ERα, possibly by preventing the ubiquitination of K302 or by promoting the ERα–calmodulin interaction. Moreover, SETD7 knockdown impairs ERα recruitment to its target genes in human breast cancer cells.^29,89^ AR also stimulates cell proliferation and anti-apoptotic responses, and it is the clinical biomarker of choice for prostate cancer diagnosis and treatment decisions.^88,90^ AR methylation at K630 by SETD7 enhances AR transcriptional activity.^36,89^ Thus, a potential role for SETD7 in the regulation of hormone-independent growth and endocrine resistance and its use as a cancer biomarker and/or as a therapeutic target should be explored.

### E2F1 and pRb

E2F1 is a transcription factor that is activated when, via CDK4/cyclin, D1 inhibits its binding partner, retinoblastoma protein (pRb). Thus, upon pRb inactivation, E2F1 promotes *CYCLIN E* transcription and progression from the G1 to S phase of the cell cycle.^91–93^ E2F1 is also activated in response to DNA damage, functioning as a G1/S checkpoint and pro-apoptotic protein.^94,95^ SETD7 methylates E2F1 at K185, which destabilizes it by enhancing E2F ubiquitination and proteosomal degradation^96^ and thus inhibits its transcriptional activity. However, others have shown that methylation of E2F1 K185 enhances E2F1-mediated apoptosis.^97^ More recently, a study by Lezina et al. confirmed that methylation of K185 attenuates E2F1 expression. However, SETD7 also enhances transcription of the E2F1 target gene *CYCLIN E* and repression of *TP53*, probably due to the formation of a regulatory loop, in which SETD7-dependent methylation and subsequent ubiquitination of E2F1 are necessary for E2F1 full transcriptional activity, but eventually lead to E2F1 degradation. Therefore, the balance between E2F1 and SETD7 expression could determine progression through the G1/S checkpoint, supporting the idea that SETD7 activity could be therapeutically targeted in E2F1-overexpressing tumors.^98^ The E2F1-negative regulator pRb is usually functionally inactivated in most human neoplasms (such as retinoblastoma, osteosarcoma, lung, breast and hepatic cancers) by mutation and/or inhibition.^99^ pRb methylation at K873 by SETD7 is required for its activity, including pRb-dependent cell cycle arrest, senescence and differentiation.^100^ SETD7 also methylates K810 of pRb, which prevents Cdk-dependent phosphorylation of pRb at adjacent amino acids. As pRb phosphorylation is necessary for the release of E2F1 and allows its transcriptional activity, pRb methylation at K810, SETD7 promotes a stable pRb/E2F1 association and consequent cell cycle arrest. This effect was found to be particularly relevant during the DNA damage response.^101^ Therefore, SETD7 may function as a tumor suppressor as well, by positively regulating pRb activity and pRb interaction with E2F1.

### Forkhead Box O3

Forkhead Box O (FoxO) transcription factors regulate cellular responses to stress, promoting the repair of the damage caused by ROS.^102^ FoxO3 is induced in response to oxidative stress to regulate cell cycle arrest, apoptosis, autophagy, metabolism and aging.^102–104^ In addition, premature follicular activation, ovarian failure and early infertility were observed in FoxO3-knockout mice.^105^ FoxO factors function as tumor suppressors in mammals.^103^ Specifically, FoxO3 interacts with ERα to inhibit its transcriptional activity and breast cancer cell proliferation.^106^ FoxO3 downregulation is associated with poor prognosis in estrogen-dependent breast cancer^103^ and low survival in late-stage colorectal cancers.^107^ SETD7 catalyzes FoxO3 methylation at K271, decreasing its stability but enhancing FoxO3-dependent activation of pro-apoptotic genes, possibly through SETD7 HMT activity. Therefore, SETD7 may be a critical regulator of the FoxO3 tumor-suppressor activity, as well as FoxO3 ability to promote longevity.^103,104,106,107^ However, SETD7 also seems to methylate K270, which has opposite effects of those provoked by K271 methylation, preventing FoxO3 from binding to the promoter of the pro-apoptotic gene *BIM* in neuronal cells and preventing cell death.^108^

### Farnesoid X receptor

Farnesoid X receptor (FXR) is a nuclear receptor activated by bile acids and regulates the transcription of genes that are crucial for bile acid homeostasis (inhibiting synthesis and secretion) and lipid, cholesterol and glucose metabolism in the liver and intestines.^109,110^ FXR also promotes liver repair/regeneration,^111^ inhibits pro-inflammatory gene expression^112,113^ and cholesterol gallstone formation^114^ and preserves the intestinal epithelial barrier.^113,115^ FXR is a tumor suppressor that has increased liver tumor incidence in FXR-knockout in mice.^115^ SETD7-dependent methylation of FXR at K206 enhances transcription of two FXR target genes, *SHP* and *BSEP*. SETD7 might stabilize FXR, facilitating FXR heterodimerization with retinoic X receptors and binding to FXR response elements, thereby promoting recruitment of FXR co-activators or inhibiting FXR interactions with co-repressors.^116^ FXR K210 and K460 may also be methylated by SETD7, although this still needs to be confirmed.^117^

### Human immunodeficiency virus transactivator

Human immunodeficiency virus transactivator (HIV-Tat) is one of the first viral proteins produced after the HIV provirus infects a cell and integrates into the host genome. Tat forms a complex with positive transcription elongation factor b (P-TEFb) and transactivation response element (TAR) RNA to promote RNA polymerase II phosphorylation by CDK9 and the efficient elongation of viral transcripts and is therefore essential for viral replication. Tat is monomethylated by SETD7 at K51. K51 is located within the arginine-rich motif (ARM), which mediates the Tat–TAR interaction and modulates Tat nuclear localization and stability.^118–120^ Recently, Ali et al. described an additional SETD7-mediated monomethylation on Tat at K71. This study also suggested that methylation of Tat at K71 and K51 are both important for the stability of Tat-TAR interaction and Tat transactivation.^121^ Therefore, SETD7 strengthens the Tat/TAR RNA/P-TEFb interaction to enhance Tat-dependent transactivation of several viral and cellular genes, contributing to viral replication and HIV-1 pathogenesis.^118–121^

### Hypoxia-inducible factor 1α

Hypoxia-inducible factor 1α (HIF-1α) is a transcriptional activator of genes that are involved in cell adaptation to low oxygen tension, altering energy metabolism and promoting angiogenesis to maintain tissue integrity and homeostasis.^122^ Under normal oxygen levels, HIF-1α is tightly regulated by the von Hippel-Lindau disease tumor suppressor (VHL) tumor suppressor, which induces ubiquitination-dependent proteasomal degradation of HIF-1α.^123^ SETD7 methylates HIF-1α at K32, which prevents K32 ubiquitination and HIF-1α degradation. Thus, SETD7 stabilizes the HIF-1α protein and stimulates HIF-1α-dependent transcription. SETD7 also monomethylates H3K4 at the promoters of HIF-1α-activated genes, suggesting that SETD7 is involved in metabolic adaptation in hypoxic cancer cells.^124,125^ By contrast, another study showed that SETD7-mediated methylation of K32 negatively regulated HIF-1α transcriptional activity by preventing its binding to DNA, an effect that was reversed by (R)-PFI-2.^126^ Therefore, additional research must be conducted to clarify the effects of SETD7 on the function of HIF-1α.

### Interferon regulatory factor 1

Interferon regulatory factor 1 (IRF1) is a transcription factor that acts as a regulator of immune responses and the cell cycle, as well as apoptosis in hematopoietic development.^127,128^ IRF1 was reported to act as a tumor suppressor, promoting cell cycle arrest or apoptosis induced by DNA damage either in cooperation with or independently of p53.^129^ SETD7 methylates IRF1 at K126. However, IRF1 might have a secondary methylation site because even after K126 mutation, weak methylation activity was still detected. Further studies should focus on determining how these modifications affect IRF1 function.^30^

### LIN28A

LIN28A is an RNA-binding protein that is expressed in embryonic stem cells (ESCs); LIN28A confers self-renewal properties and pluripotency to these cells and prevents cell differentiation.^130,131^ This effects may explain its oncogenic role because it is overexpressed in several cancers and contributes to the cancer stem cell (CSC) phenotype, as well as maintenance and growth.^132^ LIN28A promotes mRNA translation and represses pri-/pre-microRNAs maturation, such as let-7 (whose mature form promotes cell differentiation). LIN28A is predominantly localized in the cytoplasm, blocking pre-let-7 processing by Dicer.^130,131^ SETD7 methylates LIN28A at K135, which signals the localization of LIN28A to the nucleus. In addition, SETD7-mediated methylation of LIN28A at K135 enhances LIN28A stability, as well as its binding affinity to pri-let-7, maximizing the inhibition of let-7 maturation by LIN28A in the nucleus.^131^

### Methyl-CpG binding protein 2

Methyl-CpG binding protein 2 (MeCP2) is a nuclear protein that recognizes and binds methylated DNA, inhibiting gene expression by forming a complex with Sin3 and a HDAC.^133,134^ Interestingly, MeCP2 also binds hemimethylated DNA, recruiting and binding DNMT1, which transfers a methyl group to the complementary non-methylated CpG dinucleotides.^134^

MeCP2 is primarily methylated by SETD7 at K347, whereas weak methylation activity was detected even after introduction of the K347 mutation. Furthermore, using MALDI mass spectrometry, Dhayalan et al. found that two methyl groups were added to MeCP2, suggesting suggests that MeCP2 might contain a second methylation site. However, the consequences of this methylation are unknown.^30,36^

### MINT

MINT (also known as SPEN) functions as a hormone-induced transcription repressor that is involved in the regulation of the cell cycle, craniofacial development, neural cell fate and apoptosis.^135^ SETD7 was reported to strongly methylate MINT in vitro and in vivo, adding two methyl groups to MINT. Moreover, mutation of K2076 led to the total loss of methylation, suggesting that MINT is dimethylated at K2076 by SETD7.^30^ To date, the consequences of this PTM are unknown.

### NFκB-p65

NFκB is a transcription factor that has a broad range of biological functions, including inflammation, immune response, cell proliferation and apoptosis.^37^ NFκB participates in tumor immunosurveillance, which plays a role in the elimination of abnormal cells. However, dysregulation of the NFκB pathway leads to chronic inflammation, increasing the risk of developing cancer. Mutations in *NFκB* genes and/or abnormal activation of the NFκB pathway were observed in lymphomas, melanomas, leukemia, breast, prostate, colorectal and hepatic cancers and were shown to have multiple tumorigenic effects. In particular, NFκB mediates epithelial–mesenchymal transition (EMT), metastasis and angiogenesis.^136^ SETD7 methylates the nuclear NFκB-p65 subunit (also known as RelA) at K37, which restricts p65 to the nucleus and facilitates its binding to promoters of inflammatory genes, such as *TNF-α*, *MCP-1* and *IL-8*.^137^ This effect is reinforced by SETD7-driven H3K4 methylation at these promoters, enhancing p65 recruitment and stability.^37^ SETD7 also methylates K314 and K315, destabilizing p65 by promoting the ubiquitination and proteasomal degradation of DNA-bound p65.^138^ Similarly, DNA-bound p65 is also monomethylated by SETD6 at an adjacent lysine, K310. p65-K310me1 interacts with the G9A-like protein, which dimethylates H3K9 and represses the transcription of p65 target genes.^139^ Although these modifications have similar outcomes, it is not clear whether SETD7 and SETD6 work together to regulate p65. It would be interesting to explore the influence of SETD7 over NFκB and ERα, as well as AR transrepression and positive cross-talk^140,141^ as these pathways play key roles in breast and prostate cancer.

### p53

The main purpose of *p53* is to respond to cellular stress that may have its origin in DNA damage or oncogene overexpression. p53 target genes cause cell cycle arrest and apoptosis. The transcription factor p53 is downregulated or mutated in the vast majority of cancers, with a consequent impairment of its normal function. SETD7 methylation of p53 at K372 enhances its function by stabilizing p53 and inhibiting its nuclear export.^27,142^ Early studies even suggested that this PTM was essential for p53 normal activity and that the loss of this modification led to deficient p53-dependent transcriptional activation.^143–145^ However, this suggestion is not supported by recent studies, which have demonstrated that SETD7 is not necessary or sufficient for p53-dependent transactivation, suggesting that there are other molecules that act in synergy with each other and SETD7 to enhance the p53 transcriptional activity^142–144^ and that, in the absence of SETD7, can compensate its function. As *p53* mutations lead to a loss of the wild-type p53 tumor-suppressor function and act as oncoproteins,^146^ it would be interesting to explore whether SETD7 also intervenes in the regulation of p53 mutants and to develop future therapeutic strategies based on the effects of SETD7 on these proteins.

### Poly-ADP-ribose polymerase 1

Poly-ADP-ribose polymerase 1 (PARP1) is a nuclear enzyme that uses nicotinamide adenine dinucleotide (NAD^+^) to generate and transfer poly-ADP-ribose to its nuclear target proteins, such as histones, transcription factors^147,148^ or even PARP1 itself, which leads to its inactivation.^149^ PARP1 is best known as a DNA damage sensor, but it is also important for chromatin replication, transcriptional regulation, cell death,^148,150,151^ cell cycle arrest^152,153^ and cell differentiation.^154^ PARP1 also activates transcription factors that are involved in the transcription of pro-inflammatory genes.^151^ PARP1 is a SETD7 substrate and is methylated at K508. This PTM enhances PARP1 enzymatic activity under basal conditions and upon oxidative stress, as well as affects PARP1 recruitment to DNA damage sites. However, it does not impair PARP1 inhibition by auto-ADP-rybosylation. In fact, auto-ADP-rybosylation of PARP1 blocks PARP1 methylation by SETD7. These effects should be further studied to better understand whether the PARP1 enzymatic activity is modulated by SETD7-induced PARP1 stabilization, sensitization to DNA damage or increased affinity to NAD^+ 147^. Additionally, it would be interesting to identify which signaling pathways are regulated by methylated PARP1 and to determine how PARP1 methylation affects the response to PAPRP1 inhibitors, which are currently in cancer Phase II clinical trials.^155^

### P300/CBP-associated factor

P300/CBP-associated factor (PCAF) is an E3 ubiquitin ligase and acetyltransferase that is responsible for the acetylation of H3K14, H4K8 and transcription regulators, such as p53, which regulate transcriptional activation, differentiation, cell cycle arrest and apoptosis.^156–159^ PCAF also recognizes acetylated proteins, such as p300, CBP,^160,161^ H3 and H4.^162^ SETD7 methylates six different lysine residues of PCAF (K78, K89, K638, K671, K672 and K692). Although the effects of these methylations are still unknown, Masatsugu et al. discovered that methylated PCAF was localized to the nucleus.^163^

### Pancreatic and duodenal homeobox protein 1

Pancreatic and duodenal homeobox protein 1 (Pdx1) is a transcription factor that promotes differentiation of pancreatic progenitor cells^164^ and pancreatic regeneration^165,166^ and regulates β cells function, proliferation and survival,^164,167^ as well as β-cell-related gene transcription.^168^ As Pdx1 regulates insulin secretion and glucose metabolism, mutations of Pdx1 or its loss can cause diabetes. Pdx1 is also overexpressed in several cancers,^168^ such as pancreatic^169^ and gastric cancer.^170^ Pdx1 was first proposed to be a SETD7 substrate by Francis et al.^171^ Maganti et al. found that Pdx1 is methylated at K123 and K131 and that K131 is necessary for the Pdx1 transcriptional activity. They then proposed that these methylations are catalyzed by SETD7 as Pdx1 transcriptional activity is significantly increased by SETD7, an effect that is reversed by the K131 mutation. However, there is not enough evidence that shows that the observed effect is mediated by SETD7-dependent methylation of Pdx1,^172^ as previous studies suggest that H3K4 methylation by SETD7 is responsible for Pdx1 target gene transactivation.^34,171^

### PPAR-γ co-activator α

Peroxisome proliferator-activated receptor-gamma (PPAR-γ) co-activator α (PGC-1α) is a transcription co-activator that functions as a docking platform for other co-activators to activate transcription.^173^ PGC-1α is required for the regulation of mitochondrial biogenesis, energy metabolism and adaptive thermogenesis. PGC-1α is also a co-activator of steroid receptors, including ERα,^174^ and has an anti-inflammatory function in muscle tissue.^175^ SETD7-mediated methylation of PGC-1α K779 is essential for PGC-1α binding to the Mediator 1 (Med1), Med17 and Spt-Ada-Gcn5-acetyltransferase (SAGA) complexes and thus for the transcription of PGC-1α target genes.^176^

### Peroxisome proliferator-activated receptor binding protein

Peroxisome proliferator-activated receptor binding protein (PPARBP), also known as Med1, is a co-activator of the transcription machinery, which enhances the expression of RNA polymerase II transcribed genes. PPARBP regulates the cell cycle, differentiation, proliferation, apoptosis and DNA repair. PPARBP is overexpressed in breast, prostate and hepatic cancers.^177,178^ PPARBP is methylated by SETD7 at K1006; however, the functional purpose of this modification is still to be determined. Similar to MeCP2, PPARBP contains an extra methylation site, as weak methylation activity was still detected after the K1006 mutation.^30^

### Sirtuin 1

Sirtuin 1 (SIRT1) is a class III HDAC whose deacetylase activity depends on NAD^+^.^179^ SIRT1 is involved in gene silencing, the DNA damage response, cell survival and metabolism.^180–183^ SIRT1 acts either as a tumor suppressor or tumor promoter depending on the targeted protein and cancer type. For example, SIRT1 can promote or prevent cancer development and proliferation by deacetylating and inhibiting p53 or NFκB, respectively. SIRT1 also deacetylates FoxO3, promoting its ubiquitination and degradation, which impairs FoxO3-dependent transcriptional activation of tumor-suppressor genes.^183^ SIRT1 methylation at K233, K235, K236 and K238 is catalyzed by SETD7. This modification impairs SIRT1 binding to its substrates either by competing with them or by inducing a conformational change in SIRT1. For example, in response to DNA damage, SIRT1 is methylated by SETD7, which inhibits the SIRT1–p53 interaction, enhances p53 acetylation and transactivation and, consequently, leads to apoptosis.^36,179^

### Smad7

Smad7 is a negative regulator of the transforming growth factor-beta (TGFβ) signaling pathway.^184^ Smad7 also regulates other signaling pathways. For example, Smad7 induces β-catenin degradation, inhibiting the Wnt pathway.^185^ Smad7 also positively regulates the assembly of the adherens junction complex.^186^ In cancer, Smad7 can function as an oncogene, promoting pancreatic, colon, skin and lung cancer development and progression, or as a tumor suppressor, inhibiting the formation of metastasis in melanoma and breast cancer and inducing apoptosis of prostate cancer cells.^187^ SETD7 methylates Smad7 at K70,^188^ which is also acetylated by p300 to prevent Smad7 ubiquitination.^189^ Thus, p300 and SETD7-mediated modifications are mutually exclusive, with K70 methylation leading to Smad7 ubiquitination and proteasomal degradation. Moreover, SETD7 knockdown leads to a decrease in the expression of TGFβ target genes, i.e., extracellular matrix genes and genes that promote EMT.^188^

### Sex-determining region Y-related HMG box 2

During early mammalian embryonic development, the sex-determining region Y-related HMG box 2 (Sox2), which interacts with Oct4 and Nanog, activates genes involved in the maintenance of ESC pluripotency and inhibits differentiation genes.^190,191^ Sox2 is overexpressed in many types of cancer and is linked to the CSC sub-population.^192^ SETD7 methylates Sox2 at K119, which triggers its ubiquitination and proteasomal degradation and inhibits the interaction of Sox2 with other transcriptional co-activators, as well as Sox2 transcriptional activity. As a result, SETD7 overexpression promotes ESC differentiation, which can be inhibited by preventing Sox2 ubiquitination and degradation.^193^

### Serum response factor

The serum response factor (SRF) was also identified to be a SETD7 substrate.^194^ SRF binds to specific DNA regions, called CArG boxes, to regulate the expression of genes that are involved in cell growth and differentiation. In particular, SRF regulates neural and muscle development during early embryogenesis.^195^ SETD7-mediated methylation of SRF (at an unknown lysine residue) was found to increase SRF transcriptional activity and the expression of smooth muscle differentiation genes, which is further enhanced by the presence of H3K4me1 at their promoters.^194^

### Signal transducer and activator of transcription 3

Signal transducer and activator of transcription 3 (STAT3) is activated in the cytoplasm by Janus-kinase and is phosphorylated and prompted for nucleus translocation to transactivate anti-apoptotic (such as *BCL-XL*), proliferative (like *CYCLIN D1* and *C-MYC*) and inflammatory genes.^196,197^ STAT3 is constitutively activated in breast, colon, gastric, lung, head and neck, skin and prostate cancers, contributing to cancer development.^196,198^ STAT3 transcriptional function is inhibited by dimethylation at K140, which is catalyzed by SETD7 in response to IL-6 signaling, inhibiting STAT3 binding to DNA promoters.^199,200^ Therefore, SETD7 activation could be considered to be a potential approach to inhibit STAT3-dependent cancer cell proliferation.^196,197^

### Suppressor of variegation 3–9 homolog 1

Suppressor of variegation 3–9 homolog 1 (SUV39H1) is an HMT that is responsible for H3K9 trimethylation.^201^ In contrast to the H3K4me1 modification induced by SETD7, the outcome of the H3K9me3 modification induced by SUV39H1 is chromatin condensation and transcriptional repression.^8^ These two modifications are mutually exclusive.^11,44^ SUV39H1 regulates cell differentiation and represses proliferative genes expression; SUV39H1 has a tumor-suppressor role.^202^ SETD7 methylates SUV39H1 at K105 and K123, decreasing SUV39H1 methyltransferase activity and consequently H3K9me3 levels; whether simultaneous methylation of these two lysines is necessary to modulate SUV39H1 remains to be established. SETD7 also takes part in the DNA damage response, assuring the relaxation of heterochromatin by inhibiting SUV39H1 activity. When prolonged, SETD7-induced heterochromatin relaxation leads to genomic instability and to a decline in cell proliferation.^203^

### TAF10 and TAF7

TATA-box-binding protein-associated factors (TAFs) bind gene promoters and trigger the assembly of the preinitiation complex. TAFs enhance transcription by interacting with transcriptional activators and serve as readers of epigenetic marks.^204,205^ TAF10 is methylated at K189 by SETD7, which increases TAF10 affinity for RNA polymerase II and stimulates TAF10-mediated transcription. This methylation only occurs in specific promoters, as methylation-dependent transcription was only observed in a subset of genes, such as *ERα* and *ERF1* (but not *CYCLIN E* or *HPRT*).^28^ TAF7 is possibly methylated at K5 by SETD7. To date, TAF7 methylation by SETD7 has not been confirmed in vivo, and its effects remain unknown.^36,199,206^

### TTK/MPS1

Monopolar spindle 1 (MPS1, also known as TTK) phosphorylates numerous proteins that are involved in DNA damage-induced G2/M cell cycle arrest, spindle pole duplication and chromosome alignment and segregation during mitosis.^207,208^ MPS1 upregulation in cancers is associated with uncontrolled cell proliferation and increased tumor aggressiveness.^207^ MPS1 is (weakly) methylated by SETD7 at K708; however, the functional effects of this PTM still need to be established.^30^

### YAP

YAP is a transcriptional co-activator, the activity of which is inhibited upon activation of the Hippo signaling pathway by cell–cell contact.^40,209^ By interacting with transcriptional factors, YAP enhances the expression of proliferation and anti-apoptotic genes. According to Oudhoff et al., SETD7 plays a crucial role in the Hippo signaling pathway via YAP monomethylation at K494, which prevents YAP translocation to the nucleus and decreases transcription of YAP target genes. SETD7^-/-^ MEFs are more resistant to contact inhibition of proliferation than SETD7^+/+^ MEFs, which may be a consequence of the inhibition of YAP target genes in the latter case.^40^ The same authors later confirmed SETD7-dependent YAP cytoplasmic sequestration by treating MCF7 cells with (R)-PFI-2.^35^

### Yin Yang 1

Yin Yang 1 (YY1) functions as an activator or a repressor of genes that are involved in DNA repair, cell proliferation, differentiation, apoptosis and embryonic development. YY1 is overexpressed in cancer and functions as an oncogene by regulating genes that play a role in cancer development and progression, including *C-MYC* and *TP53*.^210,211^ Intriguingly, some studies also reveal that YY1 might have a tumor-suppressive role in specific cases.^211^ YY1 is methylated by SETD7 at two lysine residues, K173 and K411, which regulate the affinity of YY1 to its consensus DNA-binding elements. Thus, SETD7 is a potential regulator of YY1 transcriptional activity, promoting YY1-dependent transactivation of specific genes that are involved in cell cycle regulation and cell proliferation.^212^

### Zinc-finger DHHC domain-containing 8

Zinc-finger DHHC domain-containing 8 (ZDHHC8) is an S-acyltransferase that mediates the reversible attachment of fatty acids onto cysteine residues (S-acylation), which regulates protein solubility, attachment to membranes, intracellular distribution, folding and stability.^213,214^ ZDHHC8 loss is associated with an increased risk of schizophrenia as ZDHHC8-knockout mice exhibit a deficit in prepulse inhibition.^215^ ZDHHC8 methylation by SETD7 occurs at K300. To date, no functional effect associated with this PTM has been described.^30,36^

## SETD7 effects in cellular differentiation and proliferation

Increasing evidence suggests that SETD7 is a key player in the regulation of cell differentiation and proliferation. Multiple studies have revealed that enhancer flanking genes required for normal embryonic development frequently harbor the H3K4me1 modification to increase chromatin accessibility in uncommitted ESCs.^7,22,25,216^ During differentiation, these pre-modified (by H3K4me1) enhancers transit into an active state that is characterized by the acetylation of H3K27.^22,25,216^ These results support the hypothesis that SETD7 is required for cell differentiation. However, these studies do not clarify whether SETD7 or other H3K4 methyltransferases participate in this process, focusing only on the presence of the H3K4me1 modification. The recent literature reveals that SETD7 expression and methyltransferase activity correlate with the cell differentiation state. In fact, pluripotent cells exhibit low SETD7 levels,^63,194^ which is thought to be a result of the action of the pluripotency maintenance proteins OCT4, NANOG and SOX2, which bind to the SETD7 promoter and suppress SETD7 expression.^194^ SETD7 is upregulated during differentiation, which agrees with its observed defects in cell differentiation in cells depleted of SETD7. Studies have shown that SETD7 is indispensable for ESC differentiation because it has been suggested that methylation of H1 histone variants (specially H1.4) by SETD7 is required for H1-dependent transcriptional inhibition of *OCT4* and *NANOG*.^63^ SETD7 also promotes ESC differentiation through the methylation and consequent degradation of SOX2.^193^ Furthermore, smooth muscle cell differentiation is regulated through SETD7-mediated methylation of H3K4 and SRF, which together regulate the expression of the differentiation genes *TAGLN* and *ACTA2*.^194^ Additionally, SETD7 is essential for the differentiation of pancreatic progenitor cells in *Xenopus* and in mouse embryonic development, interacting with FOXA2 at an early phase of pancreatic development, which might result in FOXA2-mediated recruitment of SETD7 to the promoter of endodermal genes involved in pancreatic lineage fate specification, such as *PDX1*. Therefore, SETD7 is thought to regulate chromatin opening and the expression of genes involved in pancreatic differentiation through H3K4 methylation.^217^ SETD7 is required for myogenic differentiation via its interaction with myogenic differentiation protein (MyoD). This interaction facilitates SETD7 access to silenced nucleosome and subsequent H3K4 monomethylation, which leads to an increase in the MyoD affinity for the myogenic regulatory regions. In addition, SETD7 prevents the SUV39H1 association with MyoD, thus inhibiting nucleosome silencing through H3K9 methylation.^15^ By contrast, SETD7 knockdown was shown to have no effect over monocyte–macrophage differentiation or the genes involved in this process.^37^ In addition, SETD7 is also downregulated during brown adipocyte differentiation.^218^

In breast cancer cells, SETD7 inhibition was associated with a less differentiated, predominantly luminal phenotype by controlling the stability of E2F1 and DNMT1. With the downregulation of E2F1 and DNMT1, the signaling cascades that promote invasion and metastasis [such as the EGFR pathway] are prevented.^219^ SETD7 plays a role in the Hippo pathway, inducing cytoplasmic retention and inhibition of methylated YAP, which may enhance cell differentiation and inhibit proliferation. However, this hypothesis was not explored in this study and remains untested.^40^ In line with this finding, SETD7 increases the stability and transcriptional activity of ERα,^29^ a marker associated with luminal gene expression signatures.^220^ Consistent with these studies, the proliferation, migration and invasion capacity of the BT549 and MDA-MB-231 triple-negative breast cancer cell lines are increased upon SETD7 knockdown. SETD7 overexpression correlates with a decrease in the expression of the proliferation marker Ki67 in these breast cancer cells, as well as a reduced tumor size.^221^ Similar effects were observed for three gastric cancer cell lines (MKN74, MKN45 and AGS), which are thought to be mediated through H3K4 monomethylation and consequent transcription activation of tumor suppressors.^222^ In HeLa cells, cell proliferation seems to be negatively regulated by SETD7-mediated methylation and destabilization of β-catenin.^70^ By contrast, SETD7 is overexpressed in hepatocellular carcinoma and some liver cancer cell lines, which correlates with a higher risk of metastasis and recurrence, large tumor size, cell proliferation and poor differentiation.^223^

In summary, the targets of SETD7 are histones, transcription factors and chromatin-remodeling enzymes, which constitute a cross-regulated cellular network (Fig. 3). These proteins can either stimulate cell proliferation through stabilizing and enhancing the transcriptional activity of ERα, AR, PGC1α, E2F1 or YY1 or inhibiting cell proliferation through pRb stabilization, YAP cytosolic sequestration, as well as induction of β-catenin and STAT3 proteasomal degradation. The role of SETD7 in maintaining the stem cell phenotype or inducing differentiation also depends on the protein targeted. SETD7 promotes the stem cell phenotype through LIN28A and HIF1α stabilization, but promotes differentiation by inhibiting SOX2, YAP and STAT3 activity, as well as enhancing SRF function. Therefore, the opposing roles reported for SETD7 in the regulation of cell proliferation and differentiation indicate that the activity and functions of SETD7 depend on the type of tissue, type of cancer and active signaling pathways at a given time, supporting its future use in targeted therapies in stratified patient populations.

## SETD7 in ER stress

To date, little research has been carried out on this topic, with only two studies by Evans-Molina et al.^34^ and Chen et al.^224^ successfully linking ER stress to the regulation of SETD7 methyltransferase activity. The first study investigated how PPAR-γ agonists (e.g., pioglitazone) enhanced insulin synthesis and secretion and prevented islet β-cell dysfunction. Following treatment of diabetic mice with pioglitazone, an increase in islet Pdx1 levels was observed. The interaction of SETD7 with Pdx1 increased SETD7-mediated H3K4 dimethylation at the *INS1/2* and *GLU2* promoters. The authors proposed that Pdx1 may assist SETD7 transport into the nucleus via an unknown mechanism.^34^ In the second study, ER stress led to an increase in SETD7 mRNA and protein expression in the kidneys of diabetic mice. XBP1 is a transcription factor that is activated by the ER stress response to the accumulation of misfolded or unfolded proteins. XBP1 was recruited to the *SETD7* gene promoter, increasing SETD7 expression. Additionally, it was observed that SETD7 monomethylates H3K4 at the *MCP-1* promoter (which is involved in inflammation and diabetic nephropathy).^224^ Thus, these two studies support a role of SETD7 in the promotion of transcription of genes to mitigate ER stress.

## SETD7 potential in targeted interventions

The results from different cell lines and tissues show that effects mediated by SETD7 are context and tissue specific. For example, the SETD7 histone-methylase function can be inhibited to induce brown adipocyte differentiation,^218^ which may prove to be beneficial for the treatment of obesity and insulin resistance. On the other hand, H3K4me2 is needed to allow PDX1 transactivation of the *INS1/2* and *GLU2* promoters in pancreatic cells. Another example is the SETD7 potential for breast cancer treatment. Currently, triple-negative breast cancer remains difficult to treat due to a lack of targeted therapies; however, PARP1 inhibitors are promising therapeutic options.^34^ Under certain conditions, SETD7 enhances PAPR1 enzymatic activity; thus, SETD7 inhibitors could synergize with PARP1 inhibitors to treat triple-negative breast cancer. Regarding ERα-positive breast cancer, SETD7 enhances the transcriptional activity of ERα; therefore, SETD7 inhibitors could also synergize with endocrine therapy, and their applicability for treating ERα-positive/endocrine-resistant breast cancer should be investigated. However, SETD7 inhibition could enhance EMT, dedifferentiation and proliferation because its activity is necessary for the Hippo pathway and activation of tumor suppressors, including pRb; thus, use of SETD7 inhibitors would not be advisable in tumors that are dependent on EGF for growth (many of which are triple negative). Supporting this idea, the SETD7 expression levels were found to be increased in breast cancer patients with complete cancer remission in comparison with patients who suffered a recurrence.^219^ Understanding the context specificity of SETD7 can lead to substantial improvements in the way that we treat certain forms of breast cancer as the activation of SETD7 expression may be useful to prevent their transition into a CSC-like state, induce apoptosis or senescence and reduce steroid hormone receptor activity. By contrast, SETD7 is overexpressed in hepatocellular carcinoma and some liver cancer cell lines, which correlates with a higher risk of metastasis and recurrence, large tumor size, cell proliferation and poor differentiation.^223^ Taken together, these results suggest that the SETD7 tumor-suppressor or oncogenic effects are dependent on each tumor driver’s alterations.

In summary, SETD7 activation/inhibition can achieve promising results only if the right cell type and tissue are targeted, which supports the need for developing a better understanding of the SETD7 target proteins and its tissue-specific effects to stratify patients who would benefit from targeting SETD7 and to develop specific vectors to carry SETD7 agonists or antagonists.

## Conclusion

SETD7 was initially identified to be a HMT that catalyzes H3K4 methylation, promoting transcriptional activation. Recently, many additional non-histone substrates of SETD7 have been described, reinforcing the significance of SETD7 in regulating gene expression through overlapping mechanisms in multiple biological processes. The SETD7 non-histone substrates are involved in cell cycle regulation, DNA repair, gene transcription, chromatin modulation, cell proliferation and differentiation. In the case of cancer, further studies of the biological and pathological effects of SETD7 can significantly contribute to the development of novel approaches for diagnosis and targeted interventions. SETD7 is also a potential target for the treatment of several other diseases, such as diabetes, inflammatory diseases and mental disorders. Therefore, it is imperative to further evaluate the role and therapeutic potential of SETD7 in a variety of models of these diseases. Therefore, future research should focus on unraveling cellular cues to identify SETD7 protein targets in different cells and under different pathological conditions, which will allow an improved understanding of the biological effects that are regulated by SETD7 in a given system and of the overall contribution of SETD7 to pathological onset and disease progression. This information is extremely important to define tissue and cellular characteristics that would benefit from targeting SETD7 with agonist or antagonist therapies.
